# Feasibility of a Community-Based Boxing Program with Tailored Balance Training in Parkinson’s Disease: A Preliminary Study

**DOI:** 10.3390/brainsci15080858

**Published:** 2025-08-13

**Authors:** Evan V. Papa, Kathryn E. Sawyer, James M. Smoliga

**Affiliations:** 1 Department of Rehabilitation Sciences, Tufts University School of Medicine, 800 5th Ave., Seattle, WA 98104, USA; kathryn.sawyer@tufts.edu (K.E.S.); james.smoliga@tufts.edu (J.M.S.); 2 Tisch College of Civic Life, Tufts University, 163 Packard Ave., Medford, MA 02155, USA

**Keywords:** Parkinson disease, balance, falls, exercise, physical activity, boxing

## Abstract

Background/Objectives: Persons with Parkinson’s disease (PD) are at elevated risk of falling due to deficits in postural control, lower limb strength, and sensory integration. While community-based boxing programs (CBPs) have shown promise in improving strength and balance, their feasibility and potential role in addressing fall risk remain unclear. This preliminary, prospective cohort study explored the feasibility of a CBP enhanced with individualized balance training tailored to somatosensory deficits and explored early indications of potential impact on fall risk and related outcomes. Methods: Twenty individuals with mild-to-moderate PD participated in a 12-week exercise program consisting of group-based boxing, functional circuit training, and one-on-one balance training based on the Modified Clinical Test of Sensory Interaction in Balance. Self-reported falls were collected at baseline and 3 months post-intervention. Secondary outcomes included standard measures of balance, gait, and functional mobility. Results: Participants demonstrated significant improvements in balance and functional mobility including the Timed Up and Go (F(2, 40.85) = 24.83, *p* < 0.001, η^2^ = 0.580), Five Times Sit-to-Stand Test (F(2, 78.13) = 50.22, *p* < 0.001, η^2^ = 0.736), and Berg Balance Scale (F(2, 193.39) = 12.72, *p* < 0.001, η^2^ = 0.414), among others. 4 participants experienced a decrease in falls, 2 experienced an increase, and the remainder had no change. Conclusions: These preliminary findings suggest that integrating individualized balance training with a CBP is feasible and may positively influence functional mobility and balance in persons with PD. However, effects on fall reduction remain inconclusive. These results should be interpreted as exploratory and used to inform the design of future structured clinical trials.

## 1. Introduction

Falls are a significant problem in people with Parkinson’s disease (PD). Approximately 38–78% of people living with PD fall each year and a substantial proportion of these (50–86%) fall recurrently [1,2,3]. The consequences of falls can include traumatic injuries, periods of prolonged immobilization, restricted activities of daily living, and even decreased life expectancy [2,3]. An economic analysis of falls estimates that the direct medical costs of fall-related fractures in PD fallers are twice those of healthy older adults [1].

Falls in persons with PD are a multifactorial problem [4]. Factors that contribute to falls include freezing of gait, cognitive impairment, impaired anticipatory and reactive balance, previous falls, and lower limb weakness [5]. Balance, fall history, and cognitive impairment are all independently associated with falling in PD [6]. Balance is the most widespread of these domains, comprising a broad spectrum of health and disability [7]. There has been a substantial increase in the number of studies attempting to demonstrate improvements in balance, gait, and fall risk reduction through exercise [8,9,10,11]. Traditional exercise interventions, which may include aerobic conditioning, muscle strengthening, balance training, and gait training, have all demonstrated functional benefits for people with PD [9,11]. More recently, non-traditional types of community exercise programs, such as dance, tai chi, and boxing have gained considerable attention [11,12]. These interventions may offer unique benefits by incorporating elements of rhythm, coordination, and cognitive engagement. For example, a systematic review by Sharp and Hewitt [13] found that dance therapy significantly improved balance and motor impairment. Similarly, tai chi has been associated with reductions in fall risk and improvements in postural stability [14]. These approaches complement more structured training protocols and underscore the importance of multimodal and patient-centered rehabilitation strategies in PD care.

Community-based boxing programs (CBP) have gained national recognition in the United States within the PD community [8,12,15,16]. Boxing has been shown to improve balance, mobility, and endurance, while also providing a challenging cognitive component for participants as they memorize dynamic combinations and movement sequences [12,15,17]. Additionally, the social support and camaraderie associated with CBP may promote sustained adherence to exercise [18]. However, there are concerns regarding a limited amount of research and conflicting results on the benefits of CBP for people with PD [19]. While several recent studies report improvements in CBP participants’ quality of life and non-motor impairments, the data on outcomes such as balance, gait, and fall reduction are still limited [16,18]. In a 2019 systematic review of the literature on boxing for PD, Morris et al. concluded that the scientific evidence has not kept up with the rapid growth and implementation of CBP [19].

Current evidence suggests balance training, which typically includes exercises that challenge control of the body’s center of mass, has a significant ability to reduce fall rates for those with mild to moderate PD [11]. A recent randomized controlled trial (RCT) compared boxing with and without kicking techniques and found that the addition of kicking techniques provided additional benefits for balance [20]. A double-blinded RCT compared boxing with sensory focused balance exercise over a ten week period [21]. While boxing improved gait function, its effects on balance and falls were not investigated. To our knowledge, no studies have included balance training tailored to somatosensory deficits in combination with CBP to assess fall rate. There remains an urgent need to identify interventions capable of reducing falls in persons with PD. Thus, the primary purpose of this investigation was to determine the effects of a combined intervention consisting of community-based non-contact boxing and individualized balance training tailored to somatosensory deficits on the number of self-reported falls in persons with mild to moderate PD. The secondary purpose was to determine the effects of this intervention on functional mobility and balance outcomes in persons with mild to moderate PD.

## 2. Materials and Methods

A prospective, quasi-experimental cohort study was conducted. The study was approved by the Institutional Review Board at Idaho State University (approval #FY2019-205). All participants provided written informed consent. Twenty participants were consecutively enrolled in the study with recruitment occurring via local neurologists, physical therapy clinics, PD support groups, and snowball sampling. A sample size calculation was based on our preliminary data with ⍺ = 0.05, attempting to reach 84% power to detect a change in our primary aim of fall reduction with an effect size of r = 0.60 [22]. A summary of participant demographics can be seen in Table 1.

Research participants were included if they: (1) reported a diagnosis of idiopathic PD; (2) were over the age of 30 y at the time of diagnosis; (3) had a Hoehn & Yahr (H&Y) score of 1.0–3.0; and (4) had the ability to provide informed consent in accordance with Good Clinical Practice and local regulations. Participants were excluded if they had: (1) H&Y score of 4.0–5.0; (2) atypical parkinsonian syndromes due to drugs, metabolic disorders, encephalitis, or degenerative diseases; (3) the presence of definite dementia by Montreal Cognitive Assessment (MoCA < 21); (4) central or peripheral nervous system disorders other than PD; (5) myopathic disease (e.g., focal myopathy) affecting skeletal muscle structure/function; (6) severe cardiovascular disease limiting exercise abilities. All participants were instructed to continue taking their prescribed antiparkinsonian medications as usual throughout the study to reflect real-world conditions. To minimize the influence of motor fluctuations associated with medication timing (i.e., “on” and “off” periods), all testing sessions were scheduled in the morning, within one hour of participants’ typical medication intake.

The intervention was centered on a community-based boxing program; however, a key distinguishing feature of this study was the integration of personalized somatosensory balance training delivered alongside group-based boxing and functional training exercises. The intervention took place at the local YMCA, a nonprofit organization that promotes health, youth programs, and community support, and consisted of three morning sessions per week for 12 weeks, guided by certified personal trainers who were trained to work with individuals with neurologic conditions such as PD. Each session began with a 20-min warm-up consisting of breathing and stretching exercises for trunk and upper/lower extremity muscles. Participants then underwent 45 min of circuit training consisting of general balance exercises, functional training, and boxing activities. All exercises were monitored for safety and tailored to each individual’s ability to perform the task. The functional training activities included whole-body fitness and calisthenics such as lunges, jumping rope, push-ups, resistance training, and axial mobility work. The general balance training activities were given in the group included seated and standing balance, standing marches, side stepping, etc. Tailored somatosensory exercises were given individually to each person for approximately 15 min, with trainers pulling one participant at a time to receive individualized intervention according to their baseline evaluation of the Modified Clinical Test of Sensory Interaction in Balance (CTSIB-M) [24]. Consequently, approximately one-third of each training session was devoted to individual somatosensory interventions. The CTSIB-M includes four conditions, each timed for up to 30 s, yielding a maximum total score of 120 s. A lower score reflects decreased postural stability and greater difficulty maintaining balance under altered sensory conditions. In this training protocol, the focus of participants’ balance training activities emphasized the lowest performance on any one sensory component of the CTSIB-M. For example, for a low score in visual input, individualized exercise training was given such as eye tracking, visual saccades, and head turning exercises. Additional examples of specific balance activities in the various somatosensory domains can be seen in Table 2.

The boxing activities included punching heavy bags, speed bags, rapid punches, and focus mitts. Specific timing strategies or techniques that are exclusive to boxing were intentionally excluded. Participants did not make contact with each other while boxing. Participants were allowed to pace themselves during the training sessions and take rest breaks as needed. The exercise sessions ended with a 15- to 20-min cool-down emphasizing core stretching and breathing exercises.

Outcome assessments were given on three occasions: 1 week before the beginning of the training program (T0); at 6 weeks (T1); and 1 week after the completion of 12 weeks of the training program (T2). Functional mobility outcomes included: the Timed Up and Go (TUG), Functional Reach Test (FRT), and Five Times Sit-to-Stand (5-STS). Balance outcomes included: the Berg Balance Scale (BBS) and Activities-specific Balance Confidence Scale (ABC). Spatio-temporal assessments of gait parameters were collected using VSTBalance technology (VirtuSense Technologies, Peoria, IL, USA). Descriptions of assessments and evidence for reliability and validity can be found in Table 3.

Test order biases were mitigated by administering measurements in random order. The assessments were randomized at each time point (T0, T1, T2) to mitigate potential improvements due to familiarity with the order of assessments as opposed to true changes in performance. Participants were allowed to use assistive devices (cane, walker) if required for safety and if used during activities of daily living.

Falls were assessed via self-report at baseline (T0) by asking participants to record the number of falls experienced over the previous 3 months. Post-test falls were assessed via telephone surveys after 3 months (T3, a timepoint only used for falls data) of completing the training program. Falls were defined as any event resulting in a person coming to rest unintentionally on the ground or lower level, not as a result of a major intrinsic event or overwhelming hazard [41].

Repeated measures ANOVA was used to calculate mean differences on outcomes at each time-point of assessment (T0, T1, T2) for continuous data. Pairwise comparisons were calculated using Bonferroni’s correction. The Wilcoxon Signed Ranks test with exact significance was used for count data (self-reported falls). An intention-to-treat analysis was used when one participant dropped out by carrying forward their baseline score for subsequent measurements.

Effect sizes were analyzed using Partial Eta Squared (η^2^ = 0.01 small effect, η^2^ = 0.06 medium effect, η^2^ = 0.14 large effect) [42]. Pre and post differences on falls were analyzed using the Wilcoxon Signed Rank test, with the standardized effect size (r) computed as Z/√N (with N being the total number of matched-pairs included in the analysis). All analyses were conducted using the SPSS version 28 (SPSS, Inc. Chicago, IL, USA).

## 3. Results

Twenty-two potential participants were screened for inclusion with 20 meeting the inclusion criteria. Descriptive statistics including mean and standard deviation were used for participant demographics (Table 1). Individual scores for all participants can be seen in Supplementary Tables S1 and S2. One participant (PD12) withdrew during the training program because of the need to relocate to another state.

### 3.1. Functional Mobility Outcomes

**Figure 1:**
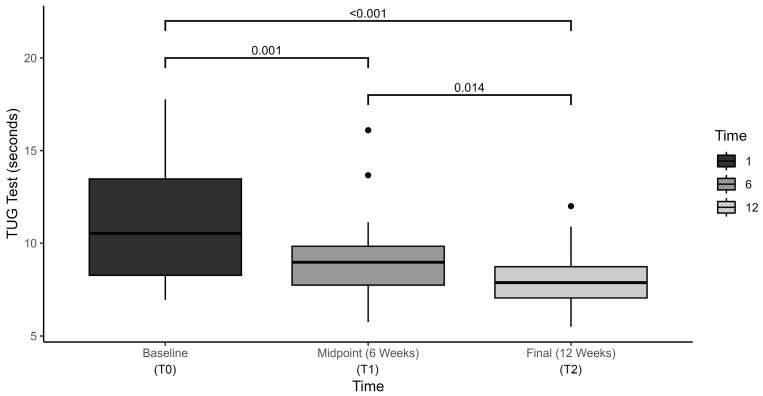
Timed Up and Go (TUG) outcomes compared at baseline, midpoint, and final assessment.

The CBP exercise intervention elicited statistically significant changes in TUG scores over time, F(2, 40.85) = 24.8, *p* < 0.001, η^2^ = 0.580, with average TUG times decreasing from (mean ± standard deviation) 11.2 ± 3.3 s at baseline, to 9.1 ± 2.5 s at six week mid-term, and to 8.1 ± 1.7 s at 12 week final assessment (Figure 1). Post hoc analysis with Bonferroni adjustment revealed that TUG scores were statistically significantly decreased from baseline to mid-term assessment (−1.9 s (95% CI, 0.75 to 3.1), *p* = 0.001), from baseline to final assessment (−2.9 s (95% CI, 1.6 to 4.2), *p* < 0.001) and from mid-term to final assessment (−1.0 s (95% CI, 0.18 to 1.8), *p* = 0.014).

**Figure 2:**
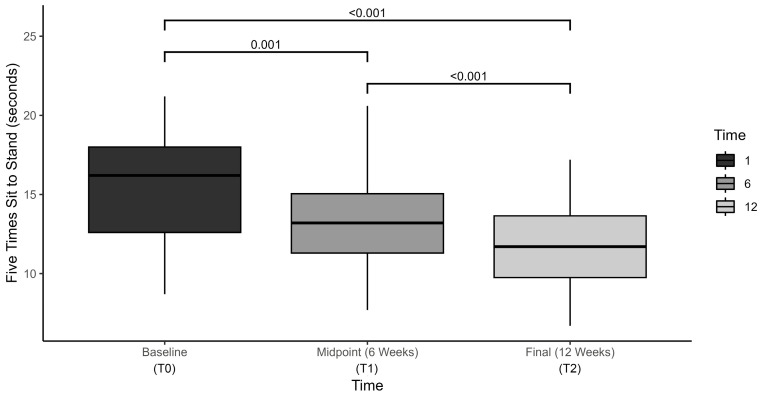
Five times sit-to-stand (5-STS) outcomes compared at baseline, midpoint, and final assessment.

Results of the 5-STS test revealed statistically significant changes over time, F(2, 78.13) = 50.2, *p* < 0.001, η^2^ = 0.736, with mean sit-to-stand times decreasing from 15.6 ± 3.4 s at baseline, to 13.4 ± 3.2 s at mid-term, and to 11.5 ± 3.1 s at final assessment (Figure 2). Post hoc analysis with Bonferroni adjustment revealed that 5-STS scores were statistically significantly decreased from baseline to mid-term assessment (−2.2 s (95% CI, 0.98 to 3.5), *p* < 0.001), from baseline to final assessment (−4.1 s (95% CI, 2.9 to 5.2, *p* < 0.001) and from mid-term to final assessment (−1.8 s (95% CI, 1.2 to 2.5, *p* < 0.001).

The exercise intervention did not elicit statistically significant changes in the FRT over time, F(2, 6.32) = 1.6, *p* = 0.215, η^2^ = 0.082, with FRT distances increasing from 8.2 ± 3.0 inches at baseline to 8.4 ± 2.9 inches at mid-term, and to 9.3 ± 2.8 inches at final assessment.

### 3.2. Balance Outcomes

**Figure 3:**
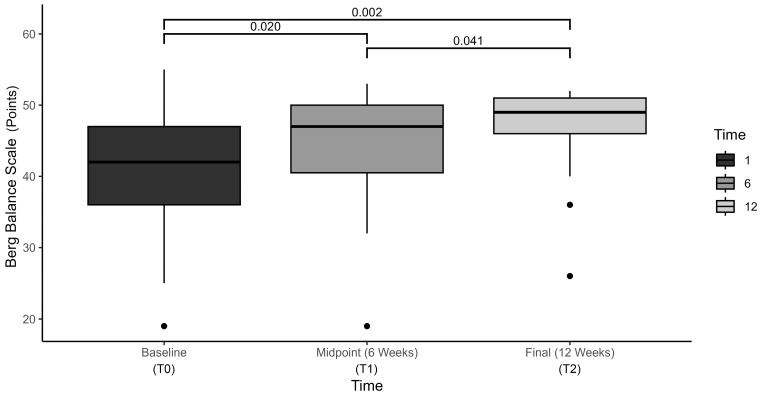
Berg Balance Scale (BBS) compared at baseline, midpoint, and final assessment.

Results of the BBS revealed statistically significant changes over time, F(2, 193.39) = 12.7, *p* < 0.001, η^2^ = 0.414, with average scores increasing from 40.5 ± 9.5 at baseline, to 44.0 ± 8.7 at mid-term, and to 46.8 ± 6.7 at final assessment (Figure 3). Pairwise comparisons demonstrated statistically significant increases at all three time points (*p* < 0.05).

**Figure 4:**
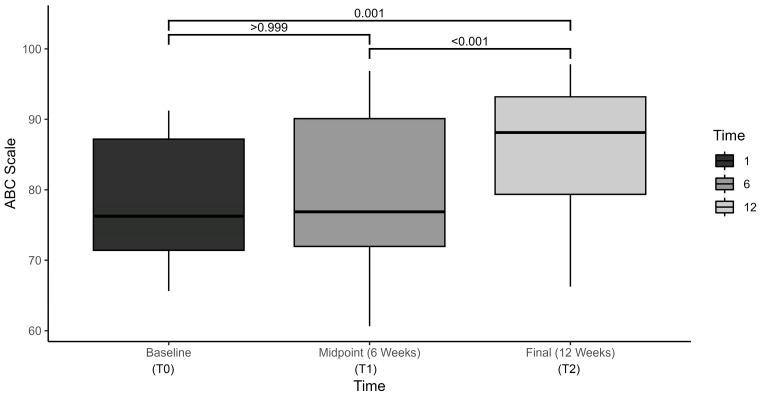
Activities-Specific Balance Confidence Scale (ABC) compared at baseline, midpoint, and final assessment.

The test of balance confidence also demonstrated statistically significant improvements over time, F(2, 290.08) = 14.2, *p* < 0.001, η^2^ = 0.442. Average ABC scores increased from 78.9 ± 8.7 at baseline, to 79.7 ± 10.8 at mid-term, and to 86.1 ± 9.5 at final assessment (Figure 4). Pairwise comparisons demonstrated statistically significant increases from baseline to final assessment only (7.1 ± 1.7, 95% CI, 2.7 to 11.6, *p* = 0.001) (Table 4).

### 3.3. Gait Parameters

Gait velocity changed significantly over time from 87.6 ± 16.8 cm/s at baseline, to 101.9 ± 21.6 cm/s at mid-term, to 100.5 ± 14.9 cm/s at final assessment (F2, 1183.93) = 10.7, *p* < 0.001, η^2^ = 0.372. Pairwise comparisons demonstrated statistically significant improvements at each time point (*p* < 0.003).

No other gait parameters (Cadence, Step time, Cycle time, and Step length) were statistically significantly changed over time.

### 3.4. Falls

**Figure 5:**
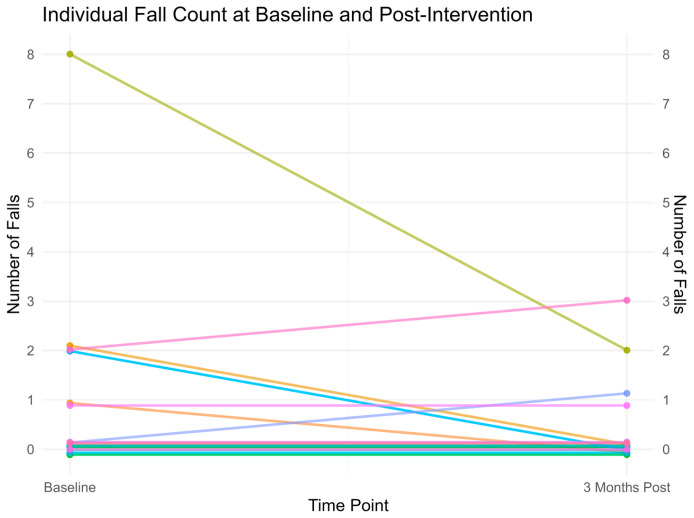
Individual fall counts at baseline and 3 months after intervention. Each line represents a single participant’s change in the number of falls from baseline to the final time point. (Slight vertical jitter was applied to each participant’s data points to reduce overplotting and improve visual separation, particularly where multiple participants had identical fall counts. Jitter is consistent within each participant and does not affect interpretation of fall values.).

4 participants experienced a decrease in falls, 2 experienced an increase, and the remainder had no change (Figure 5). A small-to-medium effect size was noted (r = 0.317).

## 4. Discussion

This preliminary feasibility study aimed to explore whether a community-based boxing program incorporating individualized somatosensory balance training could be implemented successfully and yield encouraging trends in functional outcomes for individuals with PD. The intervention was well tolerated, and participants demonstrated improvements in several balance and mobility measures. However, given the absence of a comparison group and the exploratory nature of the design, these changes cannot be attributed solely to the intervention and may reflect non-specific effects such as increased physical activity, social engagement, or placebo response. The reduction in self-reported falls was not statistically significant.

Our findings align with previous studies examining the effects of boxing on people with PD, particularly in relation to improvements in balance and mobility. Combs et al. reported enhancements in gait, balance, and quality of life in participants engaging in community-based boxing programs, though fall rates were not directly assessed [15,17]. Similarly, Moore et al. found improved balance outcomes among individuals with PD following participation in a CBP [8]. However, these studies did not incorporate individualized balance training tailored to somatosensory deficits. While our intervention combined these elements and showed promising trends in functional outcomes, the current study was not designed to isolate the specific effects of the tailored component. As such, we cannot determine whether the observed changes reflect additive benefits beyond those previously reported or are attributable to non-specific effects such as increased engagement or physical activity. Future controlled studies are needed to explore these relationships.

There is strong evidence that the basal ganglia is essential for integration of sensory information necessary to maintain balance [43,44]. In addition, ample evidence exists in clinical examinations of PD indicating declines in sensory perception [43,45]. One reason for loss of postural control in persons with PD is due to deficits in sensory integration. Specifically, the integration of somatosensory input with vestibular and visual input is compromised [46,47]. Indeed, persons with PD are known to have an overreliance on visual input for control of balance [48]. However, somatosensory focused balance training has been shown to improve balance in persons with early PD [21]. Exercises that are task-specific to individual balance impairments in persons with PD have been previously demonstrated to be effective. For example, exercises targeting poor axial mobility, difficulty with postural transitions, and slow compensatory stepping have each been shown to improve particular aspects of postural control [49,50,51]. Similarly, the specified training approach in this study resulted in improved balance for participants. Notably, a mean increase in BBS of 6.1 ± 2.8 exceeded the minimal detectable change (MDC) requirement of 5 points [25]. Additionally, large effect sizes were noted for improvements in gait speed (η^2^ = 0.372), 5-STS (η^2^ = 0.736), TUG (η^2^ = 0.580), BBS (η^2^ = 0.414) and ABC (η^2^ = 0.442).

Despite all these improvements in balance and mobility measures, self-reported falls were not statistically decreased. A clinically meaningful decrease of −9 falls occurred from pre to post intervention for the group, resulting in a small effect size (*r* = 0.317), but a translation to beyond statistical significance was not evident. This result is consistent with findings from a large systematic review and meta-analysis, demonstrating improvements in balance following exercise, but no change in fall incidence in PD [52]. Canning et al. also demonstrated improvements in balance and mobility with exercise, with an unchanged fall rate in their randomized controlled trial [53]. Consistent with Canning et al. our subgroup analysis revealed that this effect might be related to disease severity. Persons with milder PD in our study were less likely to fall than individuals with more advanced disease (e.g., persons with H&Y 1.0–2.0 accounted for 43% of the falls). The program may have exposed PD patients to greater physical activity (both directly in the CBP program, and indirectly through greater confidence) and thus more potential situations where a fall was possible—yet falls did not increase. These findings may suggest the program is safe and tolerable. However, further investigation is required to evaluate net clinical benefit.

The lack of statistically significant change in self-reported falls may also be due to a combination of small sample size, outliers in the data set, and multiple participants with no falls at baseline. Since many participants started at 0 falls, there was no room for improvement (which biases results) and there were numerous ties in pre-vs. post-differences (which limits the utility of the Wilcoxon test). Another factor that may have contributed to the lack of statistically significant reduction in fall incidence is the heterogeneity of the study sample. The cohort included both fallers and non-fallers at baseline, as well as individuals across Hoehn & Yahr stages 1.0 to 3.0. This range likely introduced variability in baseline fall risk, which may have obscured potential improvements in higher-risk individuals. Among participants with H&Y stages ≥2.0 (*n* = 13), a trend toward fall reduction was more evident. For example, two individuals (PD03 and PD05, Supplementary Table S2) reduced their fall count by 2 and 6 respectively. While others maintained zero falls, the few cases of increased fall counts were isolated and did not appear to cluster by severity. These preliminary trends suggest that individuals with moderate PD may derive more benefit in terms of fall risk, although this study was not powered for stratified subgroup analysis.

This study has several limitations. First, the study design did not incorporate a comparison group and therefore does not possess the ability to make claims regarding effectiveness of the exercise intervention. We further acknowledge that the functional improvements identified cannot be attributed solely to the boxing component, as it was part of a multi-faceted program. Efficacy of the overall program however, in regard to improvements in functional mobility, balance, and certain gait parameters, remains in effect—the natural history of PD should cause a deterioration in all of these outcomes [54]. Second, the recruitment protocol was driven by advertisement, which has a propensity to lead to a convenience sample of individuals who are motivated to exercise and interested in improving balance. The high adherence rate to the program was a strength of the study. Third, the study sample was composed of generally older, community-dwelling persons with mild-to-moderate PD, and therefore results may not be generalized beyond these demographics. Fourth, while the overall structure of the intervention was standardized, the timing of the individualized somatosensory training varied among participants within each session. Although this may have introduced minor differences in the circuit training experience, all participants received the same total duration and proportional distribution of the intervention components. This consistency in training volume and structure—rather than the specific timing or ordering—has been shown to be a strong determinant of functional outcomes, particularly in relation to strength gains [55]. Finally, there is possibility of recall bias for baseline fall incidence [56]. However, more recent evidence suggests there is a moderate level of agreement between prospective monitoring and retrospective self-reporting when it comes to categorizing individuals who have experienced falls [57], both of which were reported in this study.

Future studies should incorporate a randomized controlled design with appropriate comparison groups to better isolate the effects of specific intervention components, such as boxing or somatosensory training. Expanding recruitment strategies to include more diverse populations—including those with more advanced stages of PD, varying geographic and socioeconomic backgrounds, and individuals less inclined to engage in exercise—will improve the generalizability of findings. Additionally, the use of wearable technologies or in-home monitoring may provide more objective and continuous data on balance and fall incidence, reducing reliance on self-reported outcomes.

## 5. Conclusions

This study was the first to demonstrate the results of a community-based boxing program with specificity of individualized somatosensory balance training in self-reported falls and fall risk factors. Our results suggest that the intervention is feasible to implement and may be associated with improvements in balance, mobility, and gait parameters, though confirmatory trials are needed to evaluate effectiveness. This study also demonstrated the potential to transfer positive effects to balance confidence in activities of daily living. Fall incidents remained recalcitrant to this intervention. Additional explorations of these findings are needed with comparison groups to demonstrate real-world effectiveness.

## Figures and Tables

**Table 1:** Complete demographic data of research participants acquired at baseline (T0) evaluation.

Participant	Sex	Age	BMI	Hoehn & Yahr	UPDRS Motor	Disease Duration	Fall Status
PD01	M	67	26.5	1.0	16	0.6	NF
PD02	F	71	37.2	1.0	12	17	NF
PD03	M	63	32.6	2.0	14	0.8	NF
PD04	M	77	23.6	1.5	18	0.8	F
PD05	M	59	25.0	3.0	19	1.5	F
PD06	M	60	25.5	1.0	17	3	NF
PD07	M	84	19.2	3.0	22	1	F
PD08	M	73	24.3	1.0	13	4	NF
PD09	F	73	30.0	2.5	12	1	F
PD10	M	66	26.6	2.0	20	2	NF
PD11	F	73	27.5	2.0	15	17	NF
PD12	M	65	26.2	2.0	19	9	F
PD13	M	71	23.8	2.5	12	6	F
PD14	M	74	24.5	2.0	18	0.2	NF
PD15	M	79	35.0	3.0	29	8	F
PD16	M	71	26.6	2.0	16	7	NF
PD17	M	74	34.8	3.0	28	11	F
PD18	F	64	33.7	1.0	13	1.5	NF
PD19	F	79	23.0	2.0	13	9	NF
PD20	F	73	30.0	2.5	12	6	NF
MEAN (SD)		70.8 (6.4)	27.8 (4.6)	2.0 (0.7)	16.9 (4.9)	5.32 (5.2)	

BMI = Body Mass Index. UPDRS motor = Unified Parkinson Disease Rating Scale (motor section). Age, BMI, Disease Duration (years) = reported as Mean (SD). Hoehn & Yahr, UPDRS reported as Median (IQR). F = Faller; NF = Non-Faller (according to Timed-Up-and-Go score); cut-off scores based on Nocera, 2013 [23].

**Table 2:** Representative actions and progressions for the somatosensory components of the balance program.

CTSIB-M Balance Input	Actions	Progressions
Visual	Head turns—standing with both feet on the ground slowly turn the head to the right and left. Eye tracking—holding an object in the hand, move the hand so the eyes track the object. Saccades—standing with both feet on the ground, keep the head stable and only move the eyes, all the way to the left, then quickly move the eyes to the right.	• Progress from standing on both feet, to standing tandem, to standing on one limb. • Increase the speed of the task. • Add a cognitive task such as speaking a phrase or counting
Vestibular	Adaptation (gaze stability) exercises—participants were asked to move their heads in a yaw rotation while focusing on a stationary, hand-held target, called “X1 viewing,” and to progress to “X2 viewing,” in which the target and the head rotated in equal and opposite yaw directions. Substitution exercises—participants were instructed to make smooth pursuit eye movements towards a target before the head moves. Habituation exercises—designed to mildly provoke individual symptoms, such as walking while turning the head sideways.	• Increase the speed of the task • Quickly changing direction of eye and/or head movements • Increased speed of patient instructions • Decreasing the size of targets • Changing the color of targets
Somatosensory	The intention was to perform challenging balance exercises with focused attention on the somatosensory input for maintaining balance control. Specific activities included, heel-to-toe walking, balancing on a trampoline, balancing while seated on a ball, standing on foam or air-filled cushions, etc.	• Tandem stance and single limb balance • Adding additional air-filled cushions • Dual-tasking (adding verbal conversation, counting objects, math equations)

**Table 3:** Test description, reliability, validity, and responsiveness of measurement tools.

Measurement Tool	Construct	Evidence for Reliability	Evidence for Validity	Evidence for Responsiveness
Timed Up and Go (TUG)	Timed completion of rising from a chair, walking three meters, turning around, walking back to the chair, and sitting down.	Good test–retest reliability in PD (ICCs ≥ 0.80) [25,26] and excellent interrater reliability (r = 0.99) in PD [27]	Moderate to good convergent validity evidence in PD (correlated with the BBS (r = −0.78), fast gait speed (r = −0.69), and comfortable gait speed (r = −0.67) [28]	MDC = 4.85 s [29], also reported as MDC = 11 s [25]
Functional Reach Test (FRT)	Measurement of the maximum distance one can reach forward while standing in a fixed position. Provides a surrogate measure of dynamic balance and fall risk.	Excellent test–retest reliability in PD (ICC = 0.84) [30] and adequate intra-rater reliability (ICC = 0.74) [31]	Good predictive validity for future falls in PD [32,33]	MDC = 9 cm [31], also reported as MDC = 4.32 in patients with fall history [34], MDC = 8.07 in patients without fall history [34]
Berg Balance Scale (BBS)	A 14-item objective measure that assesses static balance and fall risk.	Excellent test–retest reliability (ICC = 0.94) [25] and excellent interrater reliability (ICC = 0.95) [35]	Excellent concurrent validity with the TUG (r = −0.78) and FRT (r = 0.50) [28]	MDC = 5 points [25]
Five Times Sit-to-Stand (5-STS)	Provides a method to quantify functional lower extremity strength and identify movement strategies to complete a transfer	Excellent test–retest reliability (ICC = 0.76) and excellent interrater reliability (ICC = 0.99) [36]	Excellent correlation with the MiniBEST test (r = 0.71) [36]	N/A
Activities Specific Balance Confidence Scale (ABC)	A self-report measure of balance confidence in performing various activities without losing balance or experiencing a sense of unsteadiness	Excellent test–retest reliability (ICC = 0.79) [29], (ICC = 0.96) [37]	Adequate concurrent validity with BBS (r = 0.505) [37]	MDC = 13 points [25] also reported as MDC = 11.12 points [29]
VSTBalance (cadence, gait speed)	Quantitative analysis of spatial and temporal gait measurements using machine vision	Good parallel forms reliability (r > 0.853) [38,39]	Excellent concurrent validity with Gait Rite (ICC > 0.971) [40]	N/A

MDC—Minimal Detectable Change.

**Table 4:** Balance, mobility, balance confidence, and gait outcomes.

Variable	T0	T1	T2	*p* Value	Effect Size
Cadence (steps/min)	104.2 (97.7–110.8)	111.0 (103.4–118.6)	110.5 (102.9–118.0)	0.054	0.150
Gait Speed (m/s)	0.88 (0.794–0.956)	1.0 (0.915–1.12)	1.0 (0.932–1.07)	<0.001	0.372
5xSTS (sec)	15.6 (13.9–17.2)	13.4 (11.8–14.9)	11.5 (10.0–13.1)	<0.001	0.736
TUG (sec)	11.0 (9.4–12.6)	9.1 (7.9–10.3)	8.1 (7.3–8.9)	<0.001	0.580
BBS (total)	40.5 (35.9–45.1)	44.0 (39.8–48.2)	46.8 (43.6–50.1)	<0.001	0.414
FRT (cm)	8.2 (6.8–9.3)	8.4 (6.9–9.8)	9.3 (7.9–10.6)	0.215	0.082
ABC (total)	78.92 (74.7–83.3)	79.71 (74.5–84.9)	86.05 (81.5–90.6)	<0.001	0.442

Data are presented as Mean (95% CI). 5xSTS = Five Times Sit-to-Stand, TUG = Timed Up and Go, BBS = Berg Balance Scale, FRT = Functional Reach Test, ABC = Activities Specific Balance Confidence Scale, T0 = Baseline, T1 = Midterm at 6 weeks, T2 = Final Measurement at 12 weeks.

## Data Availability

The raw data supporting the conclusions of this article will be made available by the authors on request.

## Supplementary Materials

The following supporting information can be downloaded at: https://www.mdpi.com/article/10.3390/brainsci15080858/s1, Table S1: Individual participant scores across time points for functional mobility, balance, and gait outcomes; Table S2: Individual fall counts before (T0) and after (T3) participation in the community-based boxing program, with baseline scores of Hoehn & Yahr stage, Fall Status, and Modified Clinical Test of Sensory Integration in Balance included.

## Abbreviations

The following abbreviations are used in this manuscript:

PD	Parkinson’s Disease
CBP	Community-based boxing programs
BMI	Body Mass Index
UPDRS	Unified Parkinson Disease Rating Scale
CTSIB-M	Modified Clinical Test of Sensory Interaction in Balance
TUG	Timed Up and Go
FRT	Functional Reach Test
5-STS	Five Times Sit-to-Stand
BBS	Berg Balance Scale
ABC	Activities-specific Balance Confidence Scale
