# Supplementary material for: Feasibility of a Community-Based Boxing Program with Tailored Balance Training in Parkinson’s Disease: A Preliminary Study
Source: Brain Sci. 2025 Aug 13;15(8):858. doi: 10.3390/brainsci15080858 (PMC12384128; doi:10.3390/brainsci15080858)
Supplement: Supplementary file 1 [file brainsci-15-00858-s001.zip › brainsci-3721247-supplementary.pdf]

**Supplementary Table S1. Individual participant scores across time points for functional mobility, balance, and gait outcomes**

| Partici-pant ID | TUG T0 (s) | TUG T1 (s) | TUG T2 (s) | 5xSTS T0 (s) | 5xSTS T1 (s) | 5xSTS T2 (s) | BBS T0 (score) | BBS T1 (score) | BBS T2 (score) | FRT T0 (cm) | FRT T1 (cm) | FRT T2 (cm) | ABC T0 (score) | ABC T1 (score) | ABC T2 (score) | Gait Speed T0 (m/s) | Gait Speed T1 (m/s) | Gait Speed T2 (m/s) |
|---|---|---|---|---|---|---|---|---|---|---|---|---|---|---|---|---|---|---|
| PD01 | 11.0 | 9.1 | 7.9 | 20.0 | 19.0 | 17.0 | 50 | 50 | 49 | 11.2 | 11.6 | 8.7 | 90.0 | 90.3 | 97.1 | 0.97 | 1.25 | 1.13 |
| PD02 | 9.7 | 6.6 | 7.6 | 12.0 | 10.0 | 7.0 | 50 | 52 | 51 | 13.3 | 8.3 | 12.4 | 90.0 | 90.6 | 88.1 | 0.94 | 0.86 | 1.10 |
| PD03 | 8.2 | 8.5 | 6.9 | 12.2 | 11.3 | 9.6 | 55 | 53 | 51 | 9.4 | 11.1 | 11.7 | 70.9 | 72.5 | 77.5 | 0.91 | 1.27 | 0.88 |
| PD04 | 12.1 | 9.0 | 10.9 | 21.2 | 20.6 | 17.2 | 46 | 49 | 49 | 6.3 | 8.5 | 8.0 | 88.1 | 87.5 | 91.5 | 1.02 | 1.06 | 0.93 |
| PD05 | 17.7 | 13.6 | 10.6 | 18.6 | 14.2 | 14.2 | 33 | 49 | 51 | 10.3 | 14.3 | 15.0 | 71.2 | 67.5 | 73.4 | 0.63 | 0.93 | 1.05 |
| PD06 | 8.7 | 7.9 | 7.0 | 16.5 | 15.4 | 11.9 | 46 | 47 | 52 | 12.0 | 9.4 | 9.6 | 78.8 | 96.8 | 97.8 | 1.00 | 1.11 | 0.87 |
| PD07 | 14.3 | 8.9 | 8.4 | 17.4 | 15.4 | 14.2 | 28 | 42 | 40 | 6.6 | 7.2 | 6.6 | 84.3 | 76.8 | 81.2 | 0.81 | 0.86 | 0.85 |
| PD08 | 11.3 | 9.9 | 7.8 | 19 | 14.7 | 12.1 | 34 | 32 | 36 | 11.9 | 4.6 | 5.2 | 71.5 | 60.6 | 66.2 | 0.69 | 0.89 | 1.02 |
| PD09 | 15.0 | 16.1 | 12.0 | 16.5 | 15.2 | 14.3 | 39 | 38 | 46 | 4.7 | 4.3 | 5.1 | 73.1 | 74.3 | 81.2 | 0.66 | 0.97 | 0.99 |
| PD10 | 7.9 | 7.5 | 6.2 | 15.2 | 14.9 | 11.7 | 51 | 51 | 51 | 9.4 | 10.1 | 10.9 | 85.6 | 85.6 | 94.2 | 1.03 | 1.05 | 1.09 |
| PD11 | 8.3 | 8.0 | 7.5 | 17.0 | 13.2 | 11.7 | 38 | 41 | 46 | 9.8 | 6.1 | 13.0 | 74.0 | 72.5 | 74.6 | 0.94 | 1.15 | 1.00 |
| PD12 | 11.4 | 11.4* | 11.4* | 12.5 | 12.5* | 12.5* | 37 | 37* | 37* | 8.2 | 8.2* | 8.2* | 75.1 | 75.1* | 75.1* | 0.72 | 0.72* | 0.72* |
| PD13 | 15.0 | 9.7 | 8.2 | 14.6 | 14.7 | 12.4 | 38 | 41 | 51 | 5.4 | 8.6 | 10.2 | 71.2 | 72.8 | 85.2 | 0.65 | 0.91 | 0.92 |
| PD14 | 10.5 | 9.1 | 9.0 | 16.2 | 12.7 | 13.1 | 39 | 40 | 41 | 5.8 | 5.03 | 7.8 | 75.3 | 83.7 | 91.3 | 0.76 | 0.66 | 0.85 |
| PD15 | 15.7 | 11.1 | 10.1 | 14.0 | 11.3 | 9.9 | 25 | 34 | 49 | 5.2 | 5.2 | 6.7 | 65.6 | 69.3 | 74.2 | 0.69 | 0.76 | 0.79 |
| PD16 | 7.03 | 6.5 | 5.5 | 12.6 | 7.7 | 6.8 | 42 | 47 | 49 | 9.4 | 12.6 | 7.9 | 91.2 | 93.7 | 95.6 | 0.96 | 1.24 | 1.14 |
| PD17 | 12.5 | 9.9 | 8.4 | 20.0 | 12.4 | 10.1 | 19 | 19 | 26 | 5.4 | 8.0 | 10.4 | 76.2 | 67.3 | 86.1 | 0.71 | 0.67 | 0.78 |
| PD18 | 7.5 | 7.3 | 7.3 | 11.7 | 9.6 | 9.07 | 42 | 49 | 49 | 5.6 | 9.2 | 8.6 | 86.2 | 89.8 | 92.1 | 1.07 | 1.43 | 1.27 |
| PD19 | 6.9 | 5.7 | 5.8 | 8.7 | 9.1 | 6.7 | 46 | 52 | 52 | 10.4 | 10.5 | 12.1 | 66.2 | 71.4 | 89.2 | 1.22 | 1.26 | 1.29 |
| PD20 | 9.4 | 8.2 | 7.0 | 12.6 | 12.2 | 10.1 | 48 | 50 | 51 | 3.5 | 4.5 | 6.2 | 90.0 | 91.0 | 97.7 | 0.91 | 0.96 | 1.01 |

TUG – Timed-Up-and-Go
5xSTS – Five-times Sit-to-Stand
BBS – Berg Balance Scale
FRT – Functional Reach Test
ABC – Activities-Specific Balance Confidence test
*data that were carried forward after participant dropout for intention-to-treat analysis

**Supplementary Table S2.**
Individual fall counts before (T0) and after (T3) participation in the community-based boxing program, with baseline scores of Hoehn & Yahr stage, Fall Status, and Modified Clinical Test of Sensory Integration in Balance included.

| Participant ID | H&Y Stage (T0) | Fall Status (T0) | CTSIB-M (T0) | Falls at T0 | Falls at T3 | Change in Falls (T3 - T0) |
|---|---|---|---|---|---|---|
| PD01 | 1.0 | NF | 90.4 | 0 | 0 | 0 |
| PD02 | 1.0 | NF | 94.3 | 1 | 0 | -1 |
| PD03 | 2.0 | NF | 78.2 | 2 | 0 | -2 |
| PD04 | 1.5 | F | 77.2 | 0 | 0 | 0 |
| PD05 | 3.0 | F | 41.0 | 8 | 2 | -6 |
| PD06 | 1.0 | NF | 88.2 | 0 | 0 | 0 |
| PD07 | 3.0 | F | 89.6 | 0 | 0 | 0 |
| PD08 | 1.0 | NF | 76.6 | 0 | 0 | 0 |
| PD09 | 2.5 | F | 92.6 | 0 | 0 | 0 |
| PD10 | 2.0 | NF | 94.5 | 0 | 0 | 0 |
| PD11 | 2.0 | NF | 87.9 | 0 | 0 | 0 |
| PD12 | 2.0 | F | 79.9 | 0 | 0* | 0 |
| PD13 | 2.5 | F | 88.4 | 0 | 0 | 0 |
| PD14 | 2.0 | NF | 92.1 | 2 | 0 | -2 |
| PD15 | 3.0 | F | 79.5 | 0 | 1 | 1 |
| PD16 | 2.0 | NF | 95.2 | 0 | 0 | 0 |
| PD17 | 3.0 | F | 10.2 | 1 | 1 | 0 |
| PD18 | 1.0 | NF | 91.0 | 0 | 0 | 0 |
| PD19 | 2.0 | NF | 90.5 | 2 | 3 | 1 |
| PD20 | 2.5 | NF | 91.0 | 0 | 0 | 0 |
| Total | | | | 16 | 7 | -9 |
| Std. Error | | | | 1.89 | 0.83 | -1.06 |

H&Y – Hoehn and Yahr stage
Fall Status - F = Faller / NF = Non-Faller (according to Timed-Up-and-Go score; cut-off scores based on Nocera, 2013)
CTSIB-M – Modified Clinical Test of Sensory Integration in Balance.
*data that were carried forward after participant dropout for intention-to-treat analysis
Note: Fall counts were self-reported for the 3 months preceding the intervention (T0) and during the final 3 months after the program concluded (T3). A reduction in fall count from T0 to T3 indicates individual improvement.
